# Supplementary material for: Evaluation and Heritability Analysis of the Seed Vigor of Soybean Strains Tested in the Huanghuaihai Regional Test of China
Source: Plants (Basel). 2023 Mar 16;12(6):1347. doi: 10.3390/plants12061347 (PMC10055562; doi:10.3390/plants12061347)
Supplement: Supplementary file 1 [file plants-12-01347-s001.zip › plants-2260343-supplementary.pdf]

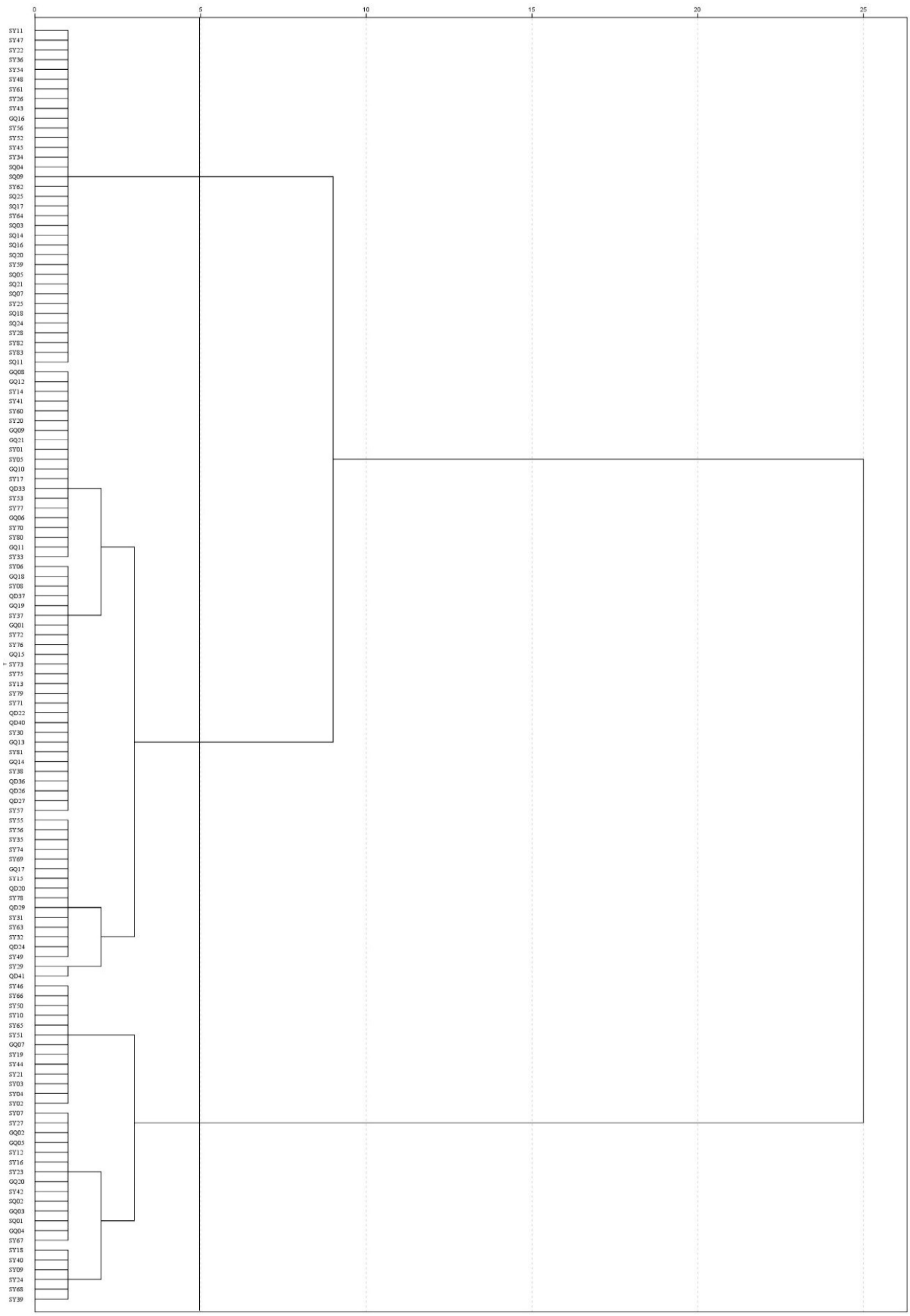

Supplementary Figure S1. Clustering analysis of seed vigor of 131 strains tested in the regional trial test in Huanghuaihai, China in 2021

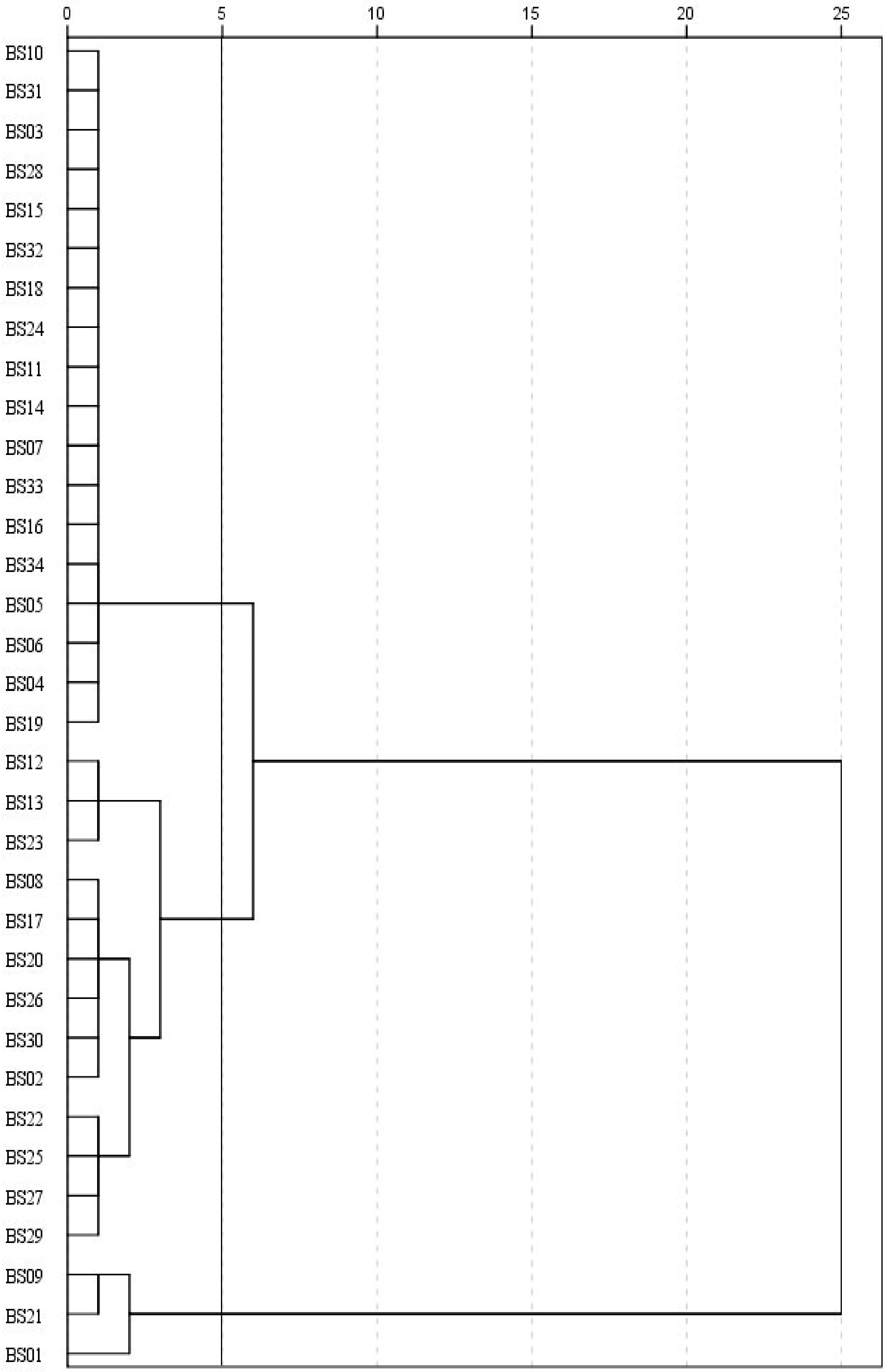

Supplementary Figure S2. Cluster analysis of seed vigor of 34 soybean strains tested in the regional trial test in Huanghuaihai, China in 2021
